# Spruce Galactoglucomannan-Stabilized Emulsions Enhance Bioaccessibility of Bioactive Compounds

**DOI:** 10.3390/foods9050672

**Published:** 2020-05-23

**Authors:** Hongbo Zhao, Kirsi S. Mikkonen, Petri O. Kilpeläinen, Mari I. Lehtonen

**Affiliations:** 1 Department of Food and Nutrition, University of Helsinki, P.O. Box 66, 00790 Helsinki, Finland; hongbo.zhao@helsinki.fi (H.Z.); Kirsi.s.mikkonen@helsinki.fi (K.S.M.); 2 Natural Resource Institute Finland, Tietotie 2, 02150 Espoo, Finland; petri.kilpelainen@luke.fi

**Keywords:** bioaccessibility, bioactive, digestion, emulsion, galactoglucomannan, polyunsaturated fatty acids

## Abstract

The increasing public awareness of health and sustainability has prompted the development of functional foods rich in health-promoting ingredients. Processing technologies and sustainable multifunctional ingredients are needed for structuring these formulations. Spruce galactoglucomannan (GGM), the main hemicelluloses in softwood cell walls, are an abundantly available, emerging sustainable food hydrocolloid that have the ability to efficiently emulsify and stabilize oil-in-water emulsions. In this study, we illustrate how this lignocellulosic stabilizer affects the digestion of polyunsaturated fatty acids (PUFAs) in vitro. A 100% decrease in the initial TAG content was observed during the in vitro digestion, suggesting that complete hydrolysis of the TAGs was achieved by the digestive enzymes. Besides, no release of mono-, di-, and oligosaccharides or phenolic compounds from GGM was detected. Our results demonstrate that the GGM-stabilized emulsion could potentially deliver lipophilic bioactive ingredients and enhance their bioaccessibility. In addition, this bio-stabilizer itself would remain stable in the upper gastrointestinal track and serve as a prebiotic for gut microbiota. We anticipate GGM to complement or even replace many of the conventional carriers of bioactive components in future health care products and functional foods.

## 1. Introduction 

Conventional technologies for utilizing wood biomass were originally developed for mainly recovering cellulose for the pulp and paper industry. Cellulose constitutes 40%–45% of wood biomass, while the remaining 55%–60% are hemicelluloses (20%–35%) and lignin [1]. Thus far, the two latter wood biomass components have the most untapped value. In order to implement a resource-wise circular economy, strategies to recover all these three constituents have recently been developed. Currently, the novel use of wood biomass is being extensively explored, with food use as one of the potential application areas. Wood biomass may provide potential multifunctional ingredients to not only improve the technological properties of food products but also introduce health-promoting compounds.

Bioactive compounds are often enriched in functional foods according to the dose-based intake of the individual. A range of lipophilic bioactive compounds, such as β-carotene, tocopherols, and polyunsaturated fatty acids, have been suggested to protect against various diseases, such as cancer, type II diabetes, myocardial infarction, atherosclerosis, and hypotension [2,3,4]. Being lipophilic, these compounds are soluble in lipid media but not miscible in an aqueous matrix. The development of functional foods or edible health care products thus requires the design of suitable delivery systems that help disperse, protect, carry, and release bioactive compounds [5,6,7]. Therefore, the food industry has been focusing on developing formulations, e.g., emulsions, to generate multiphase systems in which various bioactive ingredients may be incorporated.

Emulsification is a process in which an immiscible liquid is dispersed into another liquid to generate an emulsion [5,6,7,8]. Water and oil are the two most commonly used liquids for preparing food emulsions. Examples of these include beverages, sauces, and ice cream. Different types of emulsions are formulated by adjusting the oil-to-water ratio and the mechanical force generated by different homogenization techniques. Since emulsions are thermodynamically unstable systems, they tend to be separated into two phases. To assist their formation and increase their long-term stability, emulsifiers and stabilizers, such as small surfactants, proteins, biopolymers, and polysaccharides, are used. 

Due to consumer awareness of and negative attitudes toward synthetic food additives, emulsifiers and stabilizers from natural sources are attracting more attention. Hemicelluloses, including xylans and mannans, are among the most abundant renewable organic materials in nature [9]. However, they are still under-utilized when compared with the other major plant polysaccharides, such as cellulose and starch [10]. Methods to recover hemicelluloses from wood biomass have greatly advanced within the past decades, providing further opportunities for their utilization [11,12,13,14,15].

We have previously shown that various different galactoglucomannan (GGM)-rich lignocellulosic extracts from Norway spruce (*Picea abies*) efficiently stabilize emulsions against physical breakup and lipid oxidation [16,17,18,19,20,21,22]. GGM are the main hemicelluloses in softwoods [11,12,13]. They consist of (1→4)-linked β-d;-mannopyranosyl (Man*p*) and β-d-glucopyranosyl units with single α-d-galactopyranosyl side groups attached to the C-6 position of Man*p*. Acetylation occurs naturally in the C-2 or C-3 hydroxyl groups of the Man*p*, with a degree of approximately 0.26 [11]. The average molar mass of GGM ranges from 10,000 to 25,000 g/mol depending on the isolation method [11,12,14,15,16,17]. Lignocellulosic extracts rich in GGM and containing residues of lignin can be fractionated from Norway spruce by pressurized hot water extraction (PHWE) [14,15]. Some lignin-derived phenolic compounds and wood extractives remain with GGM after the isolation and purification process [23]. Our previous studies have shown that GGM have better emulsion stabilizing capacity than other polysaccharide-based stabilizers, such as corn fiber gum and gum Arabic [16]. Co-extracted phenolic residues introduce amphiphilic characteristics and interfacial activity to GGM. They also improve the adsorption of GGM on the lipid droplet surface and provide protection against lipid oxidation [16,17,19,20,21,22,23].

The bioaccessibility of lipophilic compounds in emulsions depends on the used emulsifiers and stabilizers. Starch and proteins are hydrolyzed during the oral and gastric phases of digestion, whereas many of the polysaccharides remain largely intact until reaching the large intestine. Plant cell wall polysaccharides are generally resistant to digestion in the upper gastrointestinal tract. Thus, if they are used as emulsion stabilizers, the changes occurring in the emulsion microstructure during digestion may be influenced and, furthermore, the bioaccessibility of the lipids affected. A decrease in the rate and extent of lipid digestion has been reported for pectin- and chitosan-stabilized emulsions, for example [6,24,25]. Pectin and chitosan are relatively large polysaccharides with a branched structure. Owing to the intermediate molar mass of GGM and their low viscosity-modifying ability [17], GGM-stabilized emulsions may exhibit unique behavior compared to other polysaccharides. Additionally, in food applications, GGM could potentially serve as a prebiotic, antioxidant and anti-inflammatory agent, as suggested by recent in vitro and in vivo studies [26,27,28,29].

With its high stabilizing capacity and health-related potential, the GGM-rich lignocellulosic extract could be a valuable candidate for the development of multiphase functional food fortified with both lipophilic and hydrophilic bioactive compounds. In this study, we reveal the potential of GGM-stabilized emulsions to deliver bioactive lipids by utilizing in vitro digestion. While GGM influences the bioaccessibility of bioactive lipids, it may also function as a prebiotic for gut microbiota. The results provide valuable insight into the use of novel, wood-derived hydrocolloids as part of a sustainable and healthy future diet.

## 2. Materials and Methods 

### 2.1. Materials

Lignocellulosic extract rich in GGM was obtained by the pressurized hot water flow-through extraction (PHWE) of Norway spruce (*Picea abies*) saw meal [14]. The extract was concentrated by ultrafiltration prior to spray-drying (Spdr-GGM) or ethanol precipitation (EtOH-GGM). Gum Arabic (Cerospray SW, C.E. Roeper GmbH, Hamburg, Germany) was used as an emulsion stabilizer for comparison. Rapeseed oil (Bunge Finland Oy, Raisio, Finland), used as lipid phase of the emulsions, was purchased from a local supermarket.

Simulated saliva fluid, simulated gastric fluid, and simulated intestinal fluid were prepared according to the INFOGEST model [30]. Bile extract (from porcine, CAS 8008-63-7), enzymes (pancreatin from porcine pancreas, CAS 8049-47-6; pepsin from porcine gastric mucosa, CAS 9001-75-6) and mucin (from porcine stomach, CAS 84082-64-4) used in the in vitro digestion model were acquired from Sigma-Aldrich (St. Louis, MO, USA). The activities of pepsin (EC 3.4.23.1), trypsin (EC 3.4.21.4), and pancreatic lipase (EC 3.1.1.3) and the concentration of bile salts were determined according to Minekus et al. [30].

Tripalmitin, which was used for the quantification of the triacylglycerols, was acquired from Sigma-Aldrich (St. Louis, MO, USA). D-glucose, D-mannose, and D-galactose, used for the quantification of mono-, di- and oligosaccharides, were acquired from Merck (Darmstadt, Germany). Phenolic acids (protocatechuic acid, ferulic acid, ellagic acid, and cya-3-glu; Extrasynthese, Genay, France) were used as external standards for the quantification of individual phenolic compounds. Solvents used in the analysis of the triacylglycerols, mono-, di- and oligosaccharides, and phenolic compounds were HPLC grade: ethanol (EtOH; 99.5%; ALTIA, Helsinki, Finland), ethyl acetate (Honeywell, Seelze, Germany), 2,2,4-trimethylpentane (iso-octane; Sigma–Aldrich, Saint Louis, MO, USA), heptane (Sigma–Aldrich, Saint Louis, MO, USA), and methanol (MeOH; Sigma–Aldrich, Saint Louis, MO, USA).

### 2.2. Emulsion Preparation

The stability of GGM against gastrointestinal digestion was investigated in Spdr-GGM- and EtOH-GGM-stabilized oil-in-water emulsions. The emulsions were prepared according to Mikkonen et al. [16]. In brief, 1 wt.% of either Spdr-GGM, EtOH-GGM, or GA was dissolved in a 25-mM citrate buffer (pH 4.5) overnight at room temperature. Then, 5 wt.% rapeseed oil was added and subsequently mixed for two minutes with an Ultra-Turrax (T-18 basic, IKA, Staufen, Germany) at 11,000 rpm to obtain coarse emulsions. The coarse emulsions were homogenized with a high-pressure homogenizer (Microfluidizer 110 Y, Microfluidics, Newton, UT, USA) for two minutes at 800 bar to obtain the final emulsion. Sodium azide (0.02 wt.%) was added to the final emulsions to inhibit microbial growth. 

### 2.3. In Vitro Digestion

The lipid delivery potential of the Spdr-GGM- and EtOH-GGM-stabilized emulsions in gastrointestinal digestion was investigated via a static INFOGEST in vitro digestion model [30] after some modifications. This model simulates the oral, gastric, and small intestinal phases of gastrointestinal digestion.

For the simulated oral phase, 5 mL of emulsion was mixed with 5 mL of simulated saliva fluid containing 150 mg of mucin. A formed oral bolus was placed on an orbital shaker for five minutes at 100 rpm and 37 °C. Mucin was included in the oral phase according to Zhang et al. [31] and Sarkar et al. [32], since it potentially destabilizes or even breaks emulsions. The use of salivary α-amylase was omitted as the studied Spdr-GGM- and EtOH-GGM-stabilized emulsions did not contain any α-linked polysaccharides.

For the gastric phase, 10 mL of simulated gastic fluid (1:1, *v*/*v*) and pepsin (200 U/mL gastric chyme) were added to the oral bolus formed during the oral phase. The pH of the formed gastric chyme was adjusted to 3.0 with hydrochloric acid. The gastric chyme was placed in an orbital shaker for two hours at 100 rpm and 37 °C. Gastric lipase was not included in the gastric phase, as it was not commercially available. The amount of pepsin was reduced from the original model, as the studied GGM fractions did not contain proteins. It was not anticipated that the proteases would influence the microstructure of the GGM-stabilized emulsions. 

For the intestinal phase, 20 mL of simulated intestinal fluid and 200 mg of bile extract (corresponding to the bile salt concentration of 6 mM) were added to the gastric chyme formed during the gastric phase. The pH of the formed intestinal chyme was adjusted to 7.0 with sodium hydroxide, after which pancreatin was added (lipase activity 1700 U/mL intestinal chyme). The gastric chime was placed in an orbital shaker for two hours at 200 rpm and 37 °C. The pH of the intestinal chyme was maintained at 7.0 for the two-hour intestinal phase through the addition of sodium hydroxide. 

In vitro digestion was repeated three times for each emulsion type (*n* = 3). Blank digestion (i.e., replacing the emulsion with a buffer solution) was performed to monitor the background various ingredients cause in the chemical analyses. The samples were protected from light throughout the experiments in order to avoid any light-induced alterations in the phenolic compounds and lipids.

After each of the three digestion phases, aliquot samples were withdrawn for the investigation of emulsion morphology and lipid release. Structural changes in the Spdr-GGM, EtOH-GGM, and GA were examined from the initial emulsion and from the intestinal chyme. The emulsion morphology was studied immediately after sampling, whereas the samples intended for chemical analyses were pretreated and stored at −20 °C. Pretreatment included termination of enzyme reactions by denaturing proteins either by the addition of ethanol (4:1, *v*/*v*) or by placing the fluid vessel in boiling water for five minutes.

### 2.4. Emulsion Morphology

The emulsion morphology was visualized before and after each digestion step by optical microscopy (Axio Scope A1, Carl Zeiss Inc., Oberkochen, Germany). The droplet size distribution was determined by static light scattering (Mastersizer 3000, Malvern Instruments, Worcestershire, United Kingdom).

### 2.5. Analysis of Triacylglycerols by HPLC-ELSD

The bioaccessibility of lipids was investigated by measuring lipid release during digestion. Triacylglycerol (TAG) content was determined at each stage of digestion. For the analysis, enzymatic reactions were terminated and simultaneously Spdr-GGM, EtOH-GGM, or GA were precipitated from a 20-mL aliquot oral bolus, gastric chyme, and intestinal chyme with 80 mL of ethanol. The released lipids were then extracted three times with 100 mL isooctane. The combined extracts were evaporated to dryness and redissolved in 25 mL of heptane for further analysis with high-performance liquid chromatography in combination with an evaporative light-scattering detector (HPLC-ELSD) [33]. TAGs were quantified using an external standard method using tripalmitin as a standard at a standard curve range of 40 to 2000 ng per injection. The results were expressed as proportions (%) to the measured initial TAG content in the emulsion.

### 2.6. Determination of Molar Mass by HPSEC-MALLS-RI

To assess the stability of Spdr-GGM, EtOH-GGM during digestion, weight-average molar mass (M_w_) was determined by size-exclusion chromatography (HPSEC) in combination with multi-angle laser light scattering (MALLS) and refractive index (RI) detection [16,17]. A 20 mL aliquot of the initial emulsion and intestinal chyme were placed in boiling water for five minutes to terminate the enzyme reactions yet not causing dramatic changes to the structures and orientation of Spdr-GGM, EtOH-GGM and GA. After the heat treatment, 1 mL of the sample was filtered through a 0.45-µm nylon syringe filter (Pall Corp., Ann Arbor, MI, USA) and diluted with a 0.1-M NaNO_3_ to a final Spdr-GGM, EtOH-GGM, or a GA concentration of 2 mg/mL. The molar mass of the samples was determined against the pullulan standards with a molar mass range of 342 to 212,000 g/mol.

### 2.7. Analysis of Monosaccharides by HPAEC-PAD

The release of mono-, di-, and oligosaccharides from Spdr-GGM, EtOH-GGM, and GA was studied to evaluate the stability of these polysaccharides during digestion. Enzyme reactions were terminated and simultaneously Spdr-GGM, EtOH-GGM, and GA were precipitated from 0.4 mL aliquot samples of the initial emulsions and intestinal chyme with 3.2 mL of ethanol. The released lipids were removed with 3 × 3.6 mL of isooctane. The remaining ethanol solution was evaporated to dryness, redissolved in 1 mL MilliQ-water, and filtered using Amicon Ultra-0.5 centrifugal filter units (Millipore, Billerica, MA, USA) at 12,000× *g* for 10 min. Mono-, di-, and oligosaccharides were separated by high-performance anion exchange chromatography coupled with pulse amperometric detection (HPAEC-PAD) [34]. Quantification was performed with an external standard method using D-glucose, D-mannose, and D-galactose as standards at a range of 0.05 to 5 µg per injection. The results were expressed as µg/g emulsion.

### 2.8. Analysis of Phenolic Compounds by UHPLC-DAD-FLD

The release of phenolic residues from Spdr-GGM, EtOH-GGM, or GA was investigated to evaluate the stability of these polysaccharides during digestion. Enzyme reactions were terminated and simultaneously Spdr-GGM, EtOH-GGM, and GA were precipitated from a 20-mL aliquot emulsion and intestinal chyme with 80 mL of ethanol [19]. The released lipids were then removed with 3 × 100 mL of isooctane. The remaining ethanol solution, containing free and ethanol-soluble phenolic residues, was evaporated to dryness and redissolved in 1 mL of MilliQ-water. The pH was adjusted to 2.0, and the phenolic compounds were extracted with 3 × 0.5 mL of ethyl acetate. The combined extracts were evaporated to dryness and redissolved in 0.2 mL of 10% methanol. The analysis of the phenolic compounds was performed by ultra-high-performance liquid chromatography coupled with ultraviolet and fluorescence detection (UHPLC-DAD-FLD) [19,20,35]. The phenolic compounds were identified based on their retention times and UV and MS spectra. The compounds were grouped into six classes and quantified as: (1) hydroxybenzoates (protocatechuic acid, 280 nm); (2) flavan-3-ols (protocatechuic acid, 280 nm); (3) hydroxycinnamates (ferulic acid, 320 nm); (4) flavonols (365 nm); (5) ellagic acids and ellagic tannins (ellagic acid, 280/365 nm); and (6) anthocyanins (cya-3-glu, 520 nm). Quantification was performed using an external standard method with a content range of 4 to 180 ng per injection. The results were expressed as µg/g emulsion.

### 2.9. Statistical Analyses

Averages and standard deviations were calculated over three replicate digestions (*n* = 3). One-way ANOVA (*p* < 0.05; IBM^®^ SPSS^®^ Statistics 24) and post-Hoc analysis with a Dunnett T3 test were performed to ascertain how the emulsions changed during digestion and how the studied emulsions differed from each other at different phases.

## 3. Results

We have previously shown that spruce-derived, hemicellulose-rich extracts function as an efficient emulsion stabilizer by providing protection against physical breakup and lipid oxidation [17,18,19,20,21,22,36]. These properties are ideal for the protection and delivery of sensitive bioactive components, such as polyunsaturated fatty acids. To ensure the bioaccessibility of lipids, they need to be accessible to hydrolyzing enzymes, namely gastric and pancreatic lipases. Large polysaccharides, such as pectin and chitosan, are known to interfere with this interaction. To understand how GGM-stabilized emulsions would behave in gastrointestinal digestion and which alterations could occur in GGM themselves, these emulsions were assessed in a static in vitro digestion model. At the moment, GGM is an emerging novel food stabilizer that has not yet been approved for food use, and, therefore, the study was conducted under in vitro conditions.

### 3.1. Lipid Release from GGM-Stabilized Emulsions

The release of lipids from Spdr-GGM-, EtOH-GGM-, and GA-stabilized emulsions was detected as changes in the TAG content. A 100% decrease in the initial content was observed during the in vitro digestion, suggesting that the TAGs were completely hydrolyzed into free fatty acids by the digestive enzymes. Both of the studied GGM fractions enabled complete lipid release from the emulsions (Table 1).

Lipid release and hydrolysis did not occur during the oral phase, as the pH was neutral and no lipid hydrolyzing enzymes were present at this stage (Table 1). The observed small changes in the TAG contents resulted from the interference of mucin during the analytical procedure.

The studied emulsions remained stable during the gastric phase, and no release of lipids was observed (Table 1). The structure formed by interfacial GGM was resistant against drastic pH changes, enabling the protection of the TAGs against acid hydrolysis. In addition, since the functionality of GGM is not based in protein residues, pepsin (i.e., protease) did not influence the emulsion microstructure and stability.

A drastic change in the content of the TAGs was observed in the intestinal phase (Table 1). GGM located at the lipid droplet interface was either efficiently replaced by other surface-active components, such as phospholipids and bile salts, or directly enabled the adsorption of pancreatic lipase on the droplet surface. This enabled the interaction between the lipase and TAGs, and, therefore, the complete hydrolysis of the TAGs was possible.

The GA-stabilized emulsions exhibited somewhat different behavior during the in vitro digestion. Like in GGM-stabilized emulsions, the TAGs were not hydrolyzed during the oral phase (Table 1). Even if the interfacial properties of GA depend on protein residues, the stability of this emulsion was not influenced by oral mucin and Ca^2+^. However, the hydrolysis of lipids was evident during the gastric phase: A 10% decrease in the TAG content was observed. Although GA forms a bulky and thick interfacial layer on the lipid droplet, providing steric support against emulsion breakup, the structure was not fully resistant against the influence of acidic pH and pepsin. Nevertheless, during the intestinal phase, the bulky structure interfered with the interaction between the pancreatic lipase and TAGs, slowing down the hydrolysis of the TAGS. After two hours of intestinal digestion, 7% of the TAGs remained intact.

### 3.2. Physical Stability of GGM Stabilized Emulsions

To detect the changes occurring in the microstructure of the GGM-stabilized emulsions during the in vitro digestion, droplet size distribution was measured, and the emulsion droplets were visualized with an optical microscope.

**Figure 1:**
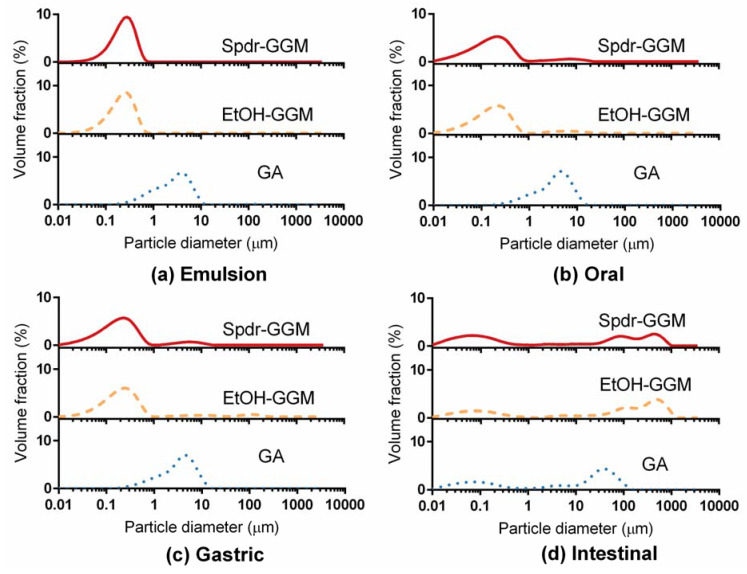
Droplet size distributions of Spdr-GGM-, EtOH-GGM-, and GA-stabilized emulsions before and after in vitro digestion: (**a**) Emulsion, (**b**) Oral phase, (**c**) Gastric phase, and (**d**) Intestinal phase.

**Figure 2:**
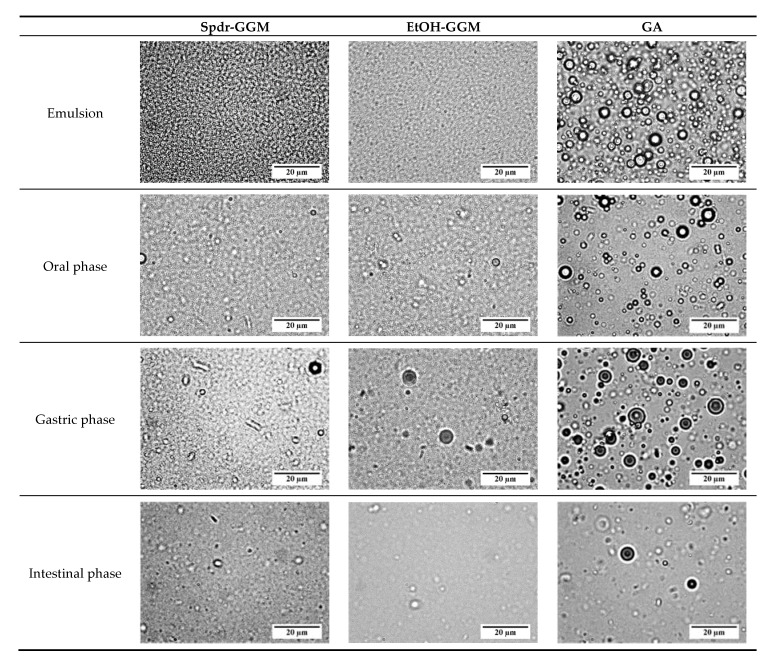
Optical microscopy images of Spdr-GGM-, EtOH-GGM-, and GA-stabilized emulsions before and after in vitro digestion.

**Figure A1:**
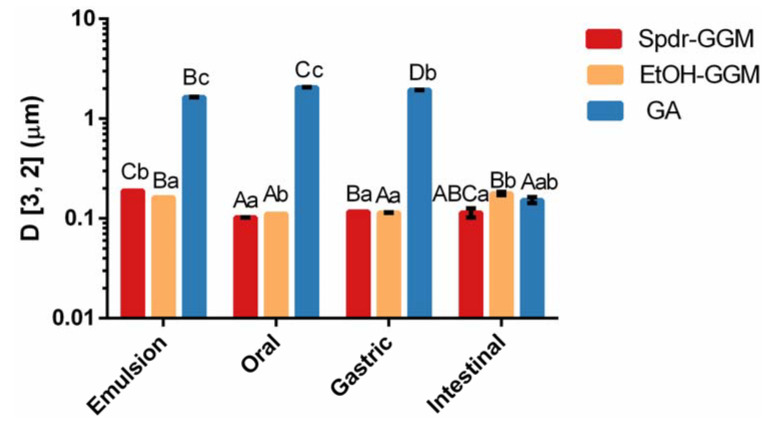
The D [3, 2] for Spdr-GGM-, EtOH-GGM-, and GA-stabilized emulsions during in vitro digestion. The capital letters compare the gastrointestinal stages, and the lowercase letters compare the emulsions (Dunnett T3 test, *p* < 0.05).

Initially, the droplets of the Spdr-GGM- and EtOH-GGM-stabilized emulsions were uniform and relatively small: D [3, 2] being 0.178 and 0.190 µm, respectively (Figure 1 and Figure 2, Appendix A Figure A1). On the contrary, in the GA-stabilized emulsion, the droplets were aggregated, and the average droplet size was 1.65 µm.

The GGM- and GA-stabilized emulsions remained physically stable, and, thus, no significant lipid release occurred during the oral and gastric phases of digestion. The droplet size distribution remained similar to that of the studied emulsions (Figure 1), and no further aggregation was observed (Figure 2). Significant changes were observed in all the studied emulsions during the intestinal phase: They fell apart during the two-hour intestinal phase, releasing hydrolyzed TAGs. Lipid droplets disappeared from the GGM-stabilized emulsions, but a few large droplets were visible in the digested GA emulsion (Figure 2). Additionally, the droplet size distribution indicated the presence of some large particles with a size between 0.1 and 1 mm. Other surface-active components, such as phospholipids and bile salts, and released free fatty acids may have interfered with the emulsion stability by replacing adsorbed GGM or GA from the droplet surface and eventually breaking down the emulsion structure. As concluded earlier, the bulky structure of GA most likely interfered with the interaction between the pancreatic lipase and TAGs, slowing down the hydrolysis of the TAGs while, at the same time, slowing down the breakup of the emulsion.

### 3.3. Stability of GGM

The stability of the studied GGM during the in vitro digestion of the GGM-stabilized emulsions was investigated by measuring the changes occurring in their molar mass, by determining the released mono-, di-, and oligosaccharides as well as by determining the released phenolic residues.

**Figure A2:**
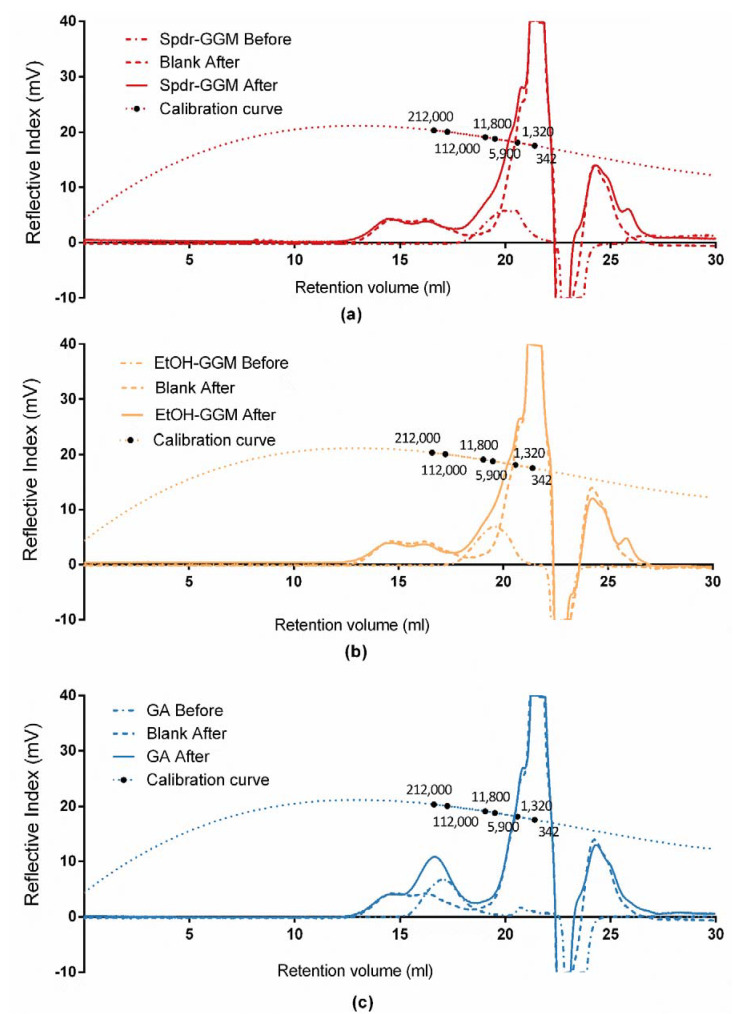
Size exclusion chromatograms of (**a**) Spdr-GGM, (**b**) EtOH-GGM, and (**c**) GA before and after in vitro digestion.

The results show that GGM and GA remained stable under the digestion conditions. The average M_w_ of the Spdr-GGM was 6000 g/mol and, for EtOH-GGM, it was 11,000 g/mol. GA was much larger than the studied GGM, with a M_w_ of 160,000 g/mol. No significant change in M_w_ occurred during the in vitro digestion (Figure A2). Spdr-GGM-, EtOH-GGM- and GA-stabilized emulsions did not contain or release any free mono-, di-, or orligosaccharides during the in vitro digestion. The total content of free phenolic residues was as low as 0.7 ± 0.1 µg/g in Spdr-GGM-stabilized emulsion, 0.1 ± 0.01 µg/g in EtOH-GGM-stabilized emulsion, and 0.5 ± 0.4 µg/g in GA-stabilized emulsion. No release of phenolic compounds was detected during the in vitro digestion. The results illustrate that GGM and GA remained stable under the gastrointestinal digestion conditions.

## 4. Discussion

The inclusion of bioactive lipophilic compounds in aqueous media enables the formulation of a large variety of food products rich in, or enriched with, these health-promoting compounds. In order to create a homogenous and stable mixture of lipophilic components and aqueous media, multifunctional stabilizers are needed. We have previously shown that GGM-rich extracts of spruce efficiently stabilize emulsions against physical breakup and inhibit lipid oxidation [17,18,19,20,21,22]. These are key features for the delivery and bioaccessibility of lipophilic compounds, for preserving bioactive components in the active state, and for avoiding the formation of adverse flavors caused by lipid oxidation. Due to their multifunctionality, GGM could be utilized in the formulation of plant-based milk products and beverages for the delivery of essential PUFAs [21].

According to the obtained results, the GGM-stabilized emulsions were able to resist the conditions during the oral and gastric phases of in vitro digestion and remained stable until the intestinal phase. Lipid droplets could be detected and visualized throughout the oral and gastric phase, and no hydrolysis of TAGs was observed. During the intestinal phase, the lipids were released and hydrolyzed by the pancreatic lipase. These observed results correlate with the stability of GGM, which retained its structure and conformation throughout the gastrointestinal digestion; in other words, the hydrolysis of GGM was not detected.

The interfacial properties of emulsifiers and stabilizers determine the formed droplets (e.g., size, charge, number, and concentration), which, in turn, influence the perceived sensory properties of emulsions [37,38,39]. Both the rheological properties and stability of emulsions influence their perceived attributes, such as creaminess and fattiness. When an emulsion breaks up during mastication, lipids form a coating on the oral surfaces, influencing, among others, flavor release and mouth feel. For example, protein-stabilized emulsions are destabilized by oral mucin, leading to early lipid release in the oral cavity and causing creamy sensations. Similarly, starch-rich formulations are degraded by oral α-amylase. The viscosity of GGM-stabilized emulsions remains very low compared to other polysaccharide-stabilized emulsions, making them potentially feasible for a wide range of food use. The viscosity of GGM solutions itself is between that of small-molecular surfactants and high molar mass macromolecular stabilizers [18]. Based on the current study, the emulsions stabilized by GGM would not break up during the oral phase through surface- or saliva-induced coalescence. These features would enable the incorporation of bioactive lipids in aqueous formulations in which a fatty mouthfeel or flavor is not desired. In addition, GGM-stabilized emulsions, being resistant against oxidation during storage and processing, lack the formation of off-flavors in lipid media [19,20,21,22]. In addition, the GGM-rich extract of spruce does not induce adverse flavors itself but has a characteristic woody flavor (unpublished data).

GGM are stable within a wide pH range and temperatures below 37 °C, and their interfacial and stabilization properties are not significantly influenced by pH [40,41]. As shown by the current data, GGM retained its structure and conformation under the gastric conditions. GGM were able to stabilize emulsions even at pH 3. When an emulsion is resistant to a low pH and gastric enzymes (namely pepsin and gastric lipase), it remains stable during the gastric phase and is able to slow down the gastric emptying [39,42]. This feature can, in turn, increase the feeling of satiety. Moreover, the slow release of homogenous gastric chyme into the intestine will be beneficial for those whose digestion is sensitive to fatty foods. In addition, when the lipid droplets remain small, a large surface area provides a large adsorption area for the pancreatic lipase. This enables efficient lipid hydrolysis and, consequently, enhances bioaccessibility. For PUFAs to get absorbed, they first need to be hydrolyzed before they can be transported to the absorption site.

The interfacial properties of GA depend on protein residues in the structure, while the amphiphilic nature of GGM depends on the coextracted lignin residues [20,22,23]. PHWE-extracted GGM are hypothesized to orientate in parallel to the droplet surface, forming a thin layer at the interface: The surface load of the GGM-stabilized emulsion is only 0.5 mg/m^2^ [20,36]. A thin layer would enable the interaction of pancreatic lipase with TAGs, resulting in an efficient hydrolysis of the TAGs and, consequently, the increased bioaccessibility of the lipids. Thus, the observed complete hydrolysis resulted from the efficient adsorption of pancreatic lipases on the droplet interface. Alternatively, other surface-active components, namely phospholipids and bile salts, efficiently replaced GGM at the surface, providing a thinner surface for the lipase to adsorb onto. While the TAGs were hydrolyzed, formed free fatty acids destabilized the emulsion structure, enabling the release of lipids. GA, having a larger Mw and more branched structure than GGM, orientates differently at the lipid droplet surface, forming a bulky and thick interface (6–10 mg/m^2^) [43]. In addition, the larger droplet size in GA emulsions decreased the contact area. The decreased surface area and increased distance between the TAGs and lipase lead to the less efficient hydrolysis of TAGs and slower lipid release. Similar behavior has been reported for other large polysaccharides [44,45]. Additionally, GA may act as a viscosity modifier and therefore reduce the mobility of lipid droplets and enzymes [46]. The bioaccessibility of lipids would thus be lower in GA-stabilized emulsions than in GGM-stabilized emulsions.

GGM being resistant against the oral, gastric, and intestinal phases of in vitro digestion indicate that they would remain intact in the gastrointestinal track and be transported to the gut intact. Gut microbiota are able to breakdown many of the ingestible dietary polysaccharides. The composition of microbial species in the gut is dependent on the dietary habits of the individual. Thus, by providing a suitable composition of ingredients that are selectively fermented (i.e., prebiotics), the composition of microbiota may be altered. In addition, fermentation metabolites are important for immune health. Recent studies have shown that softwood galactoglucomannans act as prebiotics [28,29,47]. A complete hydrolysis of β-mannans is achieved by the human gut *Firmicute Roseburia intestinalis* [47]. In addition, in an in vivo experiment on mice, softwood hemicelluloses shifted the composition of microbiota to such a position that it could potentially reduce obesity and provide cardio protection [29]. Different GGM fractions, such as Spdr-GGM and EtOH-GGM, could influence the population of gut microbiota in a different way. For example, the EtOH precipitated fraction, which contained high molecular weight oligosaccharides, produced a microbiota composition in favor of weight loss. GGM-rich extracts, which are isolated by pressurized hot water extraction, also contain lignin-carbohydrate complexes (LCC) and lignin residues [23]. Their role in gut microbiota has yet to be discovered.

For the moment, GGM is an emerging novel food stabilizer that has not yet been approved for food use. However, wood-originating polysaccharides, which are similar to the spruce GGM, namely arabinogalactan, konjac glucomannan, and guar gum, are accepted for food use. According on our recent literature review, safety hazards related to GGM are highly unlikely [48]. Thus, based on these examples, we believe that GGM has great potential as a multi-functional ingredient in future foods and health-promoting formulations.

## 5. Conclusions

The potential of GGM-stabilized emulsions to deliver bioactive compounds was evaluated through in vitro digestion. According to the obtained results, the emulsions remained stable during the oral and gastric phases but enabled efficient enzymatic hydrolysis of TAGs during the intestinal phase. Thus, GGM-stabilized emulsions are potential delivery systems of lipophilic compounds in aqueous systems, providing protection to sensitive compounds during processing and storage while enabling or even enhancing their bioaccessibility. GGM themselves remained intact during the gastrointestinal phase, but they may, in turn, function as prebiotic for gut microbiota. Due to their extensive existence and multifunctionality, GGM have great potential to be utilized not only in health-promoting formulations but also in a large variety of food applications.

## Figures and Tables

**Table 1:** Proportion of triacylglycerols (TAG %) in Spdr-GGM-, EtOH-GGM-, and GA-stabilized emulsions during in vitro digestion.

TAG %	Spdr-GGM	EtOH-GGM	GA
Initial emulsion	100 ^Aa^	100 ^Aa^	100 ^Aa^
Oral phase	88 ^Ab^ ± 2	92 ^Aa^ ± 7	96 ^Aa^ ± 1
Gastric phase	87 ^Aab^ ± 5	94 ^Aa^ ± 7	85 ^Aa^ ± 8
Intestinal phase	nd	nd	7 ^c^ ± 3

Lower case letters compare the gastrointestinal stages, and capital letters compare the emulsions (Dunnett T3 test, *p* < 0.05). EtOH-GGM = Ethanol precipitated spruce galactoglucomannan-rich extract, GA = Gum Arabic, nd = not detected, Spdr-GGM = Spray-dried spruce galactoglucomannan-rich extract.
